# Therapeutic patterns and outcomes in older patients (aged ≥ 65 years) with stage III inoperable non-small cell lung cancer (NSCLC): An investigational study from the SEER database

**DOI:** 10.1371/journal.pone.0327458

**Published:** 2025-07-03

**Authors:** Wangyan Zhong, Hang Yuan, Ting Li, Xueying Jin

**Affiliations:** 1 Department of Radiation Oncology, Shaoxing People’ s Hospital, The First Hospital of Shaoxing University, Shaoxing, Zhejiang, PR.China; 2 Department of Medical Oncology, Shaoxing People’ s Hospital, The First Hospital of Shaoxing University, Shaoxing, Zhejiang, PR.China; West China Hospital of Sichuan University, CHINA

## Abstract

**Purpose:**

Currently, optimal treatment regimens for older patients with stage III inoperable NSCLC remain unclear. The aim of this retrospective study was to investigate therapeutic patterns and survival outcomes in older patients with stage III inoperable NSCLC who received radiation therapy (RT) alone or combined with chemotherapy (CT).

**Methods:**

A retrospective analysis was conducted on 5740 patients aged ≥65 years with stage III inoperable NSCLC, using data from the Surveillance, Epidemiology, and End Results (SEER) registry (20182021). Patients treated with RT alone (n = 1077) were compared to those receiving RT + CT (n = 4663). Kaplan-Meier analysis and Log-rank tests were performed.

**Results:**

The estimated 3 years overall survival (OS) and cancer-specific survival (CSS) rates were 34.9% and 42.8%, respectively. Median OS and CSS were 21 and 28 months, respectively. Univariate analysis indicated that age, gender, T stage, pathological type and treatment option were independent prognosticators of OS and CSS. Multivariate analysis demonstrated that age, gender, T stage, N stage, and therapeutic strategy were correlated with OS, while gender, T stage, N stage and treatment option were independent prognostic factors for CSS. Subgroup analyses demonstrated that combining RT with CT improved OS in all patient subgroups, and improved CSS in all patients except those at stage N0.

**Conclusion:**

In patients aged ≥65 years with stage III inoperable NSCLC from the SEER database, treatment with RT plus CT provided longer OS and CSS compared to RT alone, except for patients with N0 stage disease.

## Introduction

Lung cancer remains a highly aggressive malignancy, and the leading cause of cancer-related mortality worldwide, with a 5-year survival rate consistently below 20%, despite therapeutic advancement [1]. Population-based analyses using the U.S. SEER registry (2013–2019) have identified significant age-dependent disparities in survival. Patients aged ≥75 years demonstrated markedly lower 5-year survival rates (8–10%) compared to younger patients (19–23% for those under 65 years), highlighting the substantial influence of advanced age on therapeutic resistance and mortality risk stratification [2]. In 2022, lung cancer fatalities exceeded the combined total deaths from colorectal, breast, and prostate cancers. Projections indicate that by 2025, approximately 500 individuals will die daily from lung cancer. Furthermore, the mortality burden of lung cancer is nearly 2.5 times greater than the combined fatalities from colorectal cancer (the second-leading cause) and pancreatic cancer (the third-leading cause), according to recent epidemiological studies [3].

Histopathological classification divides lung cancer into NSCLC (accounting for 84.7% of cases) and SCLC, with NSCLC subtypes demonstrating distinct molecular profiles influencing treatment responsiveness [4]. At diagnosis, 27.3% (95% CI: 24.8–29.9%) of NSCLC patients present with stage III disease, rendering them ineligible for curative surgery due to extensive mediastinal involvement or cardiopulmonary compromise [5,6].

For unresectable stage III NSCLC, current international guidelines recommend concurrent platinum-based chemoradiotherapy (CCRT) as the standard treatment approach, with pivotal trials reporting a median OS of 28.7 months [7,8]. However, elderly patients have systematically been excluded from landmark CCRT trials (median trial enrollment age of 61.2 years), resulting in critical evidence gaps for patients aged ≥75 years [9–12]. This therapeutic challenge is exacerbated by demographic shifts, as projections indicate that by 2035, approximately 42.6% of NSCLC cases will occur in patients aged >75 years, necessitating a reevaluation of current paradigms [1].

The updated 2024 NCCN guidelines [13] recommend CCRT followed by 12 months of consolidation therapy with durvalumab for patients with unresectable stage III NSCLC and an ECOG score ≤1, based on the PACIFIC trial protocols that demonstrated favorable outcome [14]. However, real-world analyses have revealed significant disparities in clinical practice– only 33.7% (95% CI: 29.4–38.2%) of eligible patients receive guideline-recommended treatment, with elderly patients experiencing 2.3-fold higher exclusion rates [15,16]. A Dutch multicenter, retrospective analysis involving 885 stage III NSCLC patients identified poor performance status (40%), comorbidities (35%), and advanced age (11%) as primary reasons for withholding CCRT or aggressive therapy [17]. Therefore, this study aimed to evaluate therapeutic patterns and survival outcomes in patients aged ≥65 years with stage III inoperable NSCLC.

## Materials and methods

### Database and patients’ selection

This retrospective cohort study utilized data from the National Cancer Institute’s Surveillance, Epidemiology, and End Results (SEER) 17 registry (2018−2021), representing approximately 27.8% of the US cancer population [18]. Cases were limited to histologically confirmed primary lung malignancies (ICD-O-3 codes C34.0-C34.9) with complete treatment records. Patients met the following criteria: age ≥ 65 years at diagnosis, lung cancer diagnosed as the primary malignancy between 2018 and 2021, and staging according to the EOD 8th edition. Exclusion criteria included unknown T stage, unknown N stage (NX), distant metastases (M1), unknown histological grade, and small cell histology. Therapeutic regimens for patients aged ≥65 years with locally advanced lung cancer included radiotherapy (RT) alone or combined with CT. Patients receiving no treatment were excluded from this study.

**Fig 1:**
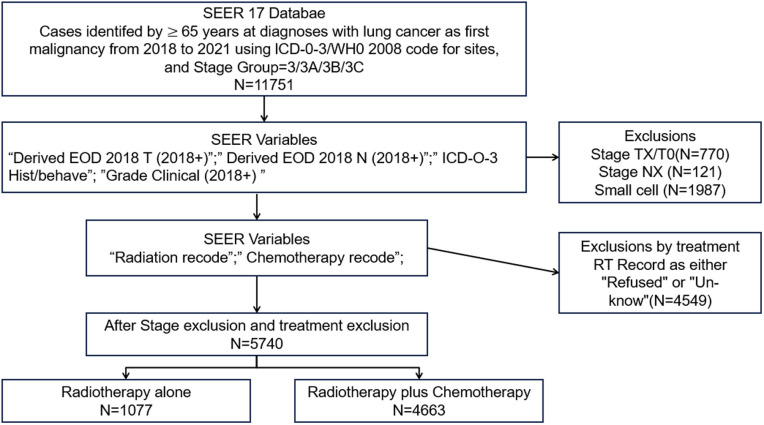
Flowchart of patient selection e aged ≥65 years diagnosed with NSCLC diagnosed from 2018 to 2021 in the SEER database, based on therapy received. Abbreviations: ICD 03: International Classification of Diseases for Oncology, 3r Edition; EOD: Extent of Disease SEER Surveillance, Epidemiology, and End Results.

Fig 1 illustrates the patient selection process. From the SEER registry, 11,751 patients with histologically confirmed lung cancer were identified. Of these, 5,740 stage III inoperable non-small cell lung cancer (NSCLC) patients aged ≥65 years who received RT with or without CT, were included for further analysis. The Institutional Review Board exempted this study from review as patient data were publicly available. Prospectively collected data included demographics, histological type, disease stage, treatment strategies, OS, and CSS.

### Statistical analyses

All statistical analyses were performed using IBM SPSS Statistics software (version 22.0) and Python programming language. OS was determined by vital status and follow-up time starting from diagnosis, while CSS was calculated using cancer-specific mortality data. Survival curves were constructed using the Kaplan-Meier method, and log-rank tests were applied to compare survival between groups. Multivariate analysis was conducted using a Cox proportional hazards regression model to identify significant prognostic factors. Hazard ratios (HRs) and corresponding 95% confidence intervals (CIs) were calculated for each factor. Statistical significance was defined as p < 0.05.

## Results

### Patient characteristics

The study cohort comprised 5,740 patients with a median age of 73.9 years (range: 65–90 years). Among these patients, 53.6% were male, 74% identified as Non-Hispanic White, 47.9% had T4 stage disease, and 58.2% were classified as N2-stage. Regarding treatment, 1,077 patients received RT alone, while 4,663 patients underwent combined RT and CT. Table 1 summarizes the cohort characteristics.

**Table 1:** Basic characteristics of patients aged ≥65 years with stage 3 inoperable NSCLC diagnosed between 2018 and 2021from the SEER Database.

Characteristics	N(%)
**Age**	
Mediate(year)	73.9
Rang (year)	65 ~ 90
65 ~ 74 years	3311(57.7%)
≥ 75 years	2429(42.3%)
**Sex**	
Male	3074(53.6%)
Female	2666(46.4%)
**Race**	
Hispanic	350(6.1%)
NHAIAN	47(0.8%)
NHAPI	391(6.8%)
NHB	690(12.0%)
NHW	4249(74.0%)
**Hist/behav**	
Squamous	2877(50.1%)
Adenocarcinoma	2475(43.1%)
Others	388(6.8%)
**T stage** ^*^	
T1	983(17.1%)
T2	1069(18.6%)
T3	1278(22.3%)
T4	2410(42.0%)
**N stage** ^*^	
N0	852(14.8%)
N1	471(8.2%)
N2	3341(58.2%)
N3	1076(18.7%)
**Therapy strategy**	
RT	1077(18.8%)
RT + CT	4663(81.2%)

Abbreviations: NHAIAN Non-Hispanic American Indian/Alaska Native; NHAPI Non-Hispanic Asia or Pacific Islander; NHB Non-Hispanic Black; NHW Non-Hispanic White; RT radiotherapy; CT chemotherapy; ^*^ The EOD 2018 staging system.

### Survival analyses

**Fig 2:**
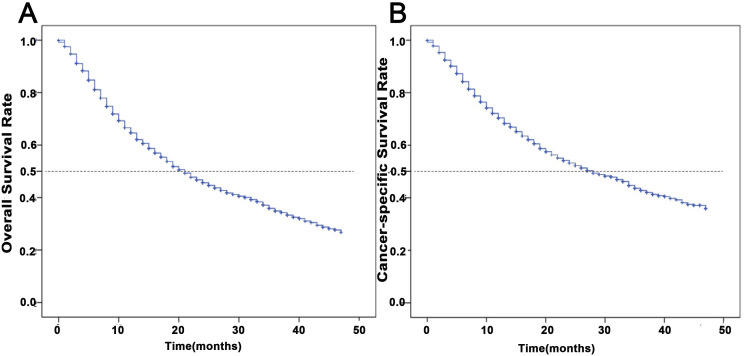
Kaplan-Meier estimates of survival among patients aged ≥ 65 years newly diagnosed with NSCLC. **(A)** OS; **(B)** CSS.

The cohort demonstrated a median OS duration of 15 months (range: 0–47 months). Comparative survival analysis revealed 1-year OS and CSS rates of 64.6% and 70.4%, respectively, declining to 34.9% and 42.8% at 3 years. Median OS and CSS differed notably at 21 and 28 months, respectively (Fig 2).

**Fig 3:**
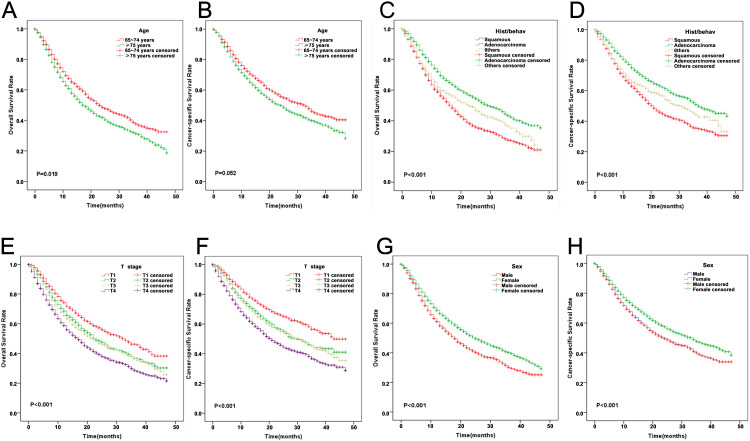
Kaplan-Meier estimates of survival among patients aged ≥ 65years newly diagnosed with NSCLC, stratified by various factors. **(A)** OS by age; **(B)** CSS by age; **(C)** OS by T stage; **(D)** CSS by T stage; **(E)** OS by Histological Pathology; **(F)** CSS by Histological Pathology; (G) OS by gender; **(H)** CSS by gender.

Age-stratified analysis showed progressively poorer outcomes with advancing age. Patients aged 65–74 years exhibited significantly higher 1-year OS (67.3% vs. 60.9%) and 3-year OS (37.6% vs. 31.2%) compared to those ≥75 years (P = 0.019; Fig 3A), while CSS was numerically higher but did not reach statistical significance (P = 0.052; Fig 3B). Median OS was notably longer in the 65–74 age group (23 months,95% CI: 21.35–24.65) compared to the older group (18 months,95% CI: 16.60–19.39; P < 0.001, S1 Table ). Similarly, median CSS was 33 months (95% CI: 30.36–35.64) for those aged 65–74 years and 24 months (95% CI: 21.71–26.29) for patients aged ≥75 years (P < 0.001, S2 Table).

Histopathological subtype significantly influenced survival outcomes. Patients with adenocarcinoma showed superior survival. One-year OS rates for squamous cell carcinoma, adenocarcinoma, and other NSCLC subtypes were 58.7%, 72.0%, and 61.9% respectively, declining to 27.4%, 43.3%, and 36.3% at 3 years (P < 0.001; Fig 3C). CSS showed a similar pattern (P < 0.001; Fig 3D) Median OS was 17 months (95% CI: 15.97–18.03) for squamous cell carcinoma, 30 months (95% CI: 27.50–32.50) for adenocarcinoma, and 23 months (95% CI: 18.24–27.76) for other NSCLC subtypes (P < 0.001, S1 Table). Median CSS showed similar trends: 21 months (95% CI: 19.55–22.46) for squamous cell carcinoma, 37 months (95% CI: 33.29–40.71) for adenocarcinoma, and 31 months (95% CI: 24.79–37.21) for other NSCLC subtypes (P < 0.001, S2 Table). T-stage demonstrated strong prognostic value, with survival decreasing progressively with higher T-stage. For OS, 1-year survival rates declined from 74.1% (T1) to 58.7% (T4), and 3-year rates decreased from 46.0% to 28.7% (P < 0.001, Fig 3E). CSS analysis reflected the same trend (P < 0.001, Fig 3F). Median OS durations were 32 months (95% CI: 27.60–36.40) for T1, 23 months (95% CI: 20.49–25.51) for T2, 22 months (95% CI: 19.21–24.79) for T3, and 17 months (95% CI: 15.67–18.33) for T4 (P < 0.001, S1 Table). Median CSS durations were 43 months (95% CI: 39.08–46.92) for T1, 30 months (95% CI: 26.07–33.93) for T2, 31 months (95% CI: 26.90–35.10) for T3, and 21 months (95% CI: 19.26–22.74) for T4 (P < 0.001, S2 Table).

A significant sex-based survival difference emerged, with female patients demonstrating better OS and CSS than male patients (P < 0.001; Fig 3G–3H).

**Fig 4:**
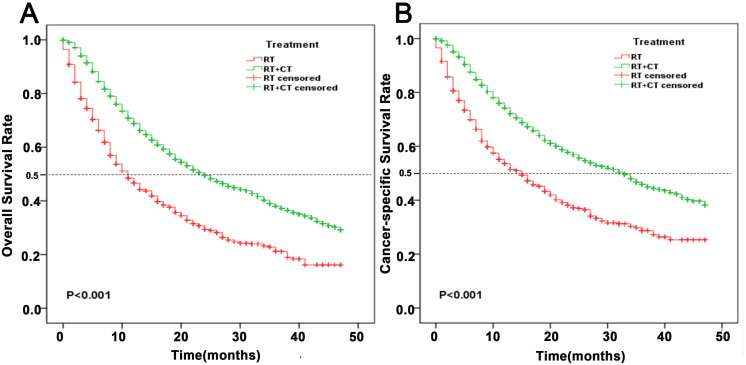
Kaplan-Meier estimates of survival among patients aged ≥65 years newly diagnosed with NSCLC treated with RT alone versus RT combined with CT. **(A)** OS; **(B)** CSS. Abbreviations: RT: radiotherapy; CT: chemotherapy.

Analysis of therapeutic modalities showed substantial survival benefits from combined RT + CT to RT alone. Patients receiving RT + CT demonstrated superior 1-year OS (68.8% vs. 44.6%) and CSS (74.3% vs. 53.5%), advantages that persisted at 3 years (OS: 38.1% vs. 21.2%; CSS: 45.9% vs. 28.7%; P < 0.001, Fig 4A–4B).

### ldentification of prognostic factors

Through comprehensive prognostic evaluation, we identified age, gender, pathological type, T-stage, N-stage, and therapeutic regimen were identified as candidate prognostic variables. Univariable Cox regression analysis revealed age, gender, pathological type, T-stage, and therapeutic strategy as significant prognostic factors for both OS and CSS (Table 2).

**Table 2:** Results of univariable analysis for both OS and CSS among newly diagnosed NSCLC patients aged ≥ 65 years in the SEER-17 database.

Characteristics	1-year OS	3-year OS	P	1-year CSS	3-year CSS	P
**Age**						
65 ~ 74 years	67.3	37.6	< 0.001	72.4	45.2	< 0.001
≥ 75 years	60.9	31.2		67.7	39.4	
**Sex**						
Male	61	30.2	< 0.001	67.6	38.8	< 0.001
Female	68.8	40.2		73.6	47.1	
**Hist/behav**						
Squamous	58.7	27.4	< 0.001	65.1	35.5	< 0.001
Adenocarcinoma	72	43.3		77.2	50.7	
Others	61.9	36.3		67.4	44.4	
**T stage** ^*^						
T1	74.1	46	< 0.001	80.2	56.4	< 0.001
T2	68.4	36.8		73.6	44	
T3	65.4	36.5		72.1	44.1	
T4	58.7	28.7		64.2	36	
**N stage** ^*^						
N0	65.8	34	0.882	70.9	44.4	0.998
N1	63.6	31		70.2	40	
N2	64.4	35.2		70.3	42.7	
N3	64.7	36.6		70.6	43.2	
**Therapy strategy**						
RT	46.6	21.2	< 0.001	53.5	28.7	< 0.001
RT + CT	68.8	38.1		74.3	45.9	

Abbreviations: RT radiotherapy; CT chemotherapy; OS overall survival; CSS Cancer-specific survival; ^*^ The EOD 2018 staging system. Hist/behave: pathological type.

**Fig 5:**
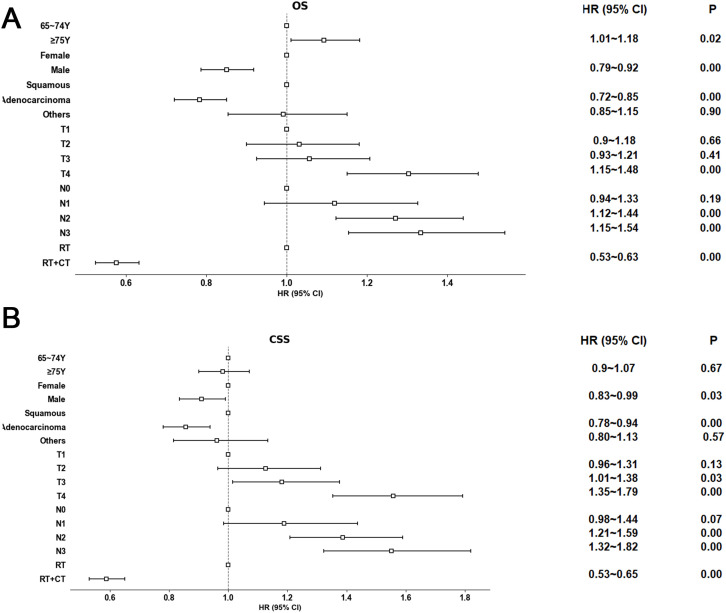
Weighted multivariable Cox proportional hazards regression analysis for OS and CSS; RT radiotherapy; CT chemotherapy; OS overall survival; CSS Cancer-specific survival.

Multivariate Cox regression analysis demonstrated that combined RT and CT was significantly associated with prolonged OS and CSS, compared to RT alone (OS HR = 0.58, 95% CI: 0.53–0.63, P < 0.001, Fig 5A; CSS HR = 0.59, 95% CI: 0.53–0.65, P < 0.001, Fig 5B). Furthermore, patients ≥75 years exhibited poorer OS compared to those aged 65−74 years (HR = 1.09, 95% CI: 1.01–1.18, P = 0.03). However, no significant difference in CSS was observed between these age groups (HR = 0.98, 95% CI: 0.90–1.07, P = 0.67, Fig 5). Male patients showed better OS and CSS than female patients (OS HR = 0.85, 95% CI: 0.79–0.92, P < 0.001, Fig 5A; CSS HR = 0.91, 95% CI: 0.83–0.99, P = 0.03, Fig 5B), suggesting a survival advantage for males.

### Subgroup analysis

To identify patients aged ≥65 years with newly diagnosed stage III inoperable NSCLC who would most benefit from combined RT and CT, a subgroup analysis was conducted. Combined RT and CT demonstrated robust survival benefits across the entire cohort. Multivariate analyses showed statistically significant improvements in OS associated with RT + CT across all evaluated prognostic factors: age (p < 0.001), sex (p < 0.001), T-stage (p < 0.001), and nodal involvement (N1-3, p < 0.001). A statistically significant survival benefit was also observed in patients with N0 disease (p = 0.036), though the magnitude was modest compared to other groups, suggesting potential biological differences in early nodal-stage tumors. This therapeutic synergy extended to CSS, where RT + CT provided significant survival advantages for age (p < 0.001), sex (p < 0.001), T-stage (p < 0.001), and N1-3 disease (p < 0.001). In contrast, CSS improvement in N0 patients did not reach statistical significance (p = 0.16), possibly indicating biological resistance in node-negative tumors. The discrepancy between OS and CSS outcomes in N0 disease warrants further investigation into tumor microenvironment characteristics and mechanisms of treatment resistance (Table 3).

**Table 3:** Subgroup analysis of both OS and CSS among patients aged ≥65 years with newly diagnosed stage III inoperable NSCLC treated with RT alone or RT + CT in the SEER-17 database.

Characteristics	1-year OS		3-year OS		P	1-year CSS		3-year CSS		P
	RT	RT + CT	RT	RT + CT		RT	RT+ CT	RT	RT+ CT	
**Age**										
65 ~ 74 years	44.6	70.4	21.3	39.7	< 0.001	51.4	75.2	28.7	47.2	< 0.001
≥ 75 years	47.7	66.2	21.1	35.2	< 0.001	54.8	72.8	28.8	43.5	< 0.001
**Sex**										
Male	45.4	64.5	17.4	33	< 0.001	53.5	70.7	27.4	41.2	< 0.001
Female	48	73.8	24.8	43.9	< 0.001	53.8	78.4	29.8	51.2	< 0.001
**Hist/behav**										
Squamous	39.3	63.6	13	30.9	< 0.001	46.2	69.7	17.9	39.1	< 0.001
Adenocarcinoma	57.3	75	30.3	46	< 0.001	64	79.9	38.2	53.2	< 0.001
Others	43.6	66.5	31.6	38.3	0.001	51.7	71.4	49.2	44.9	0.008
**T stage** ^*^										
T1	64.4	75.8	27.3	49.1	< 0.001	68.6	82	38.6	59.4	< 0.001
T2	45.8	72.4	20.4	39.6	< 0.001	52	77.4	26.4	46.9	< 0.001
T3	41	69.8	14	40.5	< 0.001	49.1	76	19.9	48.2	< 0.001
T4	44.3	63.3	22.1	30.9	< 0.001	51.4	68.3	30	38.1	< 0.001
**N stage** ^*^										
N0	60.9	68.7	32.7	34.6	0.036	69	72.2	43.6	44.8	0.16
N1	40	69.9	10	37.9	< 0.001	42.8	77.6	14.2	48	< 0.001
N2	42.8	68.5	18.1	38.4	< 0.001	49.4	74.2	24.5	46	< 0.001
N3	35.3	69.2	22.7	39.1	< 0.001	43.8	74.5	31.3	45.5	< 0.001

Abbreviations: RT radiotherapy; CT chemotherapy; SEER Surveillance, Epidemiology, and End Results; OS overall survival; CSS cancer-specific survival; ^*^ The EOD 2018 staging system.

## Discussion

To our knowledge, optimal therapeutic strategies for geriatric patients (≥65 years) with inoperable stage III NSCLC remain undefined due to historical exclusion of this population from pivotal clinical trials. Recently, neoadjuvant and perioperative chemoimmunotherapy have expanded into the stage IIIA and even IIIB settings. This study focused on patients aged ≥65 years (median age 72 years) with inoperable stage III NSCLC, whereas contemporary trials of neoadjuvant or perioperative chemoimmunotherapy predominantly enroll younger, surgically eligible patients (median age 63 years in the NADIM II trial) [19]. Older adults are frequently excluded from intensive regimens (e.g., neoadjuvant dual immunotherapy) due to increased comorbidities (e.g., COPD, cardiovascular disease) and diminished functional reserves [20]. This population-based analysis systematically evaluated 17 SEER registries to assess treatment patterns and survival outcomes among community-dwelling elderly patients (≥65 years) newly diagnosed with stage III inoperable disease. The findings confirmed a survival advantage for RT + CT compared to RT alone.

Univariate and multivariate analyses, demonstrated that RT plus CT significantly improved OS and CSS compared to RT alone. Additionally, age ≥ 75 years was associated with poorer OS and CSS compared to ages 65–74 years. Notably, age ≥ 80 years emerged as a potential adverse prognostic factor for both outcomes, with progressively declining survival rates observed across age groups (65–74, 75–79, and ≥80 years). However, given the relatively small proportion of patients aged ≥80 years (<10%) and the limited subgroup size (<42.3% of those aged ≥75 years, Table 1), the statistical power to detect significant differences was constrained, necessitating further validation in larger patient cohorts. Contrary to established observations that female patients generally have better prognoses, our results indicated that male gender correlated with improved OS and CSS. This discrepancy likely arises from biologically appropriate but clinically excessive treatment de-escalation in elderly females, as highlighted by Zimmermann et al. [21]. Although dose reductions aim to mitigate toxicity, they may disproportionately reduce therapeutic efficacy in this subgroup. Subgroup analyses indicated that combining RT and CT improved OS across all stage III inoperable NSCLC patients, and improved CSS for all patients except those at stage N0. Notably, although combined therapy demonstrates survival benefits, its application in elderly patients requires careful consideration of competing risks. Treatment-related toxicities, such as myelosuppression and renal impairment, may increase non-cancer mortality. This could reduce gains in OS despite preserved CSS benefits from effective tumor control.

The clinical imperative for geriatric-specific protocols is underscored by demographic realities: in the U.S., 70% of NSCLC diagnoses occur after age 65, with 37% in patients ≥75 years [3,22]. While theJCOG0301 trial demonstrated the feasibility of carboplatin-based chemoradiation in septuagenarians (median OS 22.4 months) [23],real-world implementation remains suboptimal, with fewer than 40% of eligible elderly patients receiving CCRT compared to 65% of younger counterparts [24,25]. This disparity likely arises from concerns regarding toxicity tolerance and the underuse of geriatric assessment tools [26]. Encouragingly, the LOGIK1902 phase II trial specifically targeting elderly NSCLC (median age 76 years) reported a median OS of 25.5-month using weekly carboplatin-paclitaxel [9], closely aligning with our institutional cohort’s 21-month OS and supporting the need for adapted therapeutic approaches This study demonstrated that in real-world practice, CCRT achieved a 3-year OS rate of 38.1% in older adults with stage III NSCLC. The PACIFIC trial redefined first-line therapy for inoperable stage III NSCLC by establishing durvalumab consolidation following CCRT as standard care, achieving a 3-year OS rate of 57%, suggesting a potential 19% absolute survival benefit compared to our observed CCRT outcomes [27]. However, this theoretical benefit must be contextualized: only 32% of SEER patients aged ≥65 years met the key eligibility criteria for the PACIFIC trial (performance status 0–1, no autoimmune disease) [28]. To minimize treatment protocol heterogeneity, patients diagnosed with stage III unresectable NSCLC between 2018 and 2021 were specifically enrolled. This period reflected broad clinical adoption of the CCRT-durvalumab sequence, thus reducing variability in multimodal therapies that could confound survival outcomes. Real-world studies report that elderly patients receiving durvalumab experience a 23% incidence of grade ≥3 pneumonitis (vs. 3.4% in PACIFIC) [29], potentially diminishing survival benefits. Furthermore, 28% of older patients initiated durvalumab more than 42 days after radiotherapy (compared to PACIFIC’s 1–42day window), possibly reducing treatment efficacy [30]. A 2023 SEER-Medicare analysis found that adding durvalumab to CCRT increased the 3-year OS rate to 43.2% in patients aged ≥65 years [31], demonstrating a 5.1% real-world survival benefit over our baseline CCRT outcomes. For the 41% of older adults ineligible for durvalumab due to comorbidities or frailty [32], our observed CCRT outcomes remain the best available survival estimates. To address this evidence gap, machine learning models integrating SEER variables (e.g., age, Charlson Comorbidity Index, radiation dose) should be developed to predict which geriatric subgroups derive the greatest benefit from consolidation therapy, thus enabling precision-based treatment allocation.

Moreover, adverse factors such as poor performance status, comorbidities, decreased organ function, and limited social support affect tolerance to CCRT in elderly patients. These individuals are frequently considered unsuitable for aggressive treatment due to their frailty [33]. RTOG 9410 documented increased grade ≥3 esophagitis (absolute risk increase of 10%) and hematologic toxicity (increase of 15%) among elderly subgroups [34]. However, Dutch registry data challenge assumptions of age-based toxicity identifying ECOG status ≥2 (OR=1.73) rather than chronological age as the primary risk determinant [35]. This discrepancy between trial-reported and real-world toxicity profiles emphasizes the need for comprehensive geriatric assessment (CGA)-guided treatment algorithms, as proposed in recent ASCO guidelines [36].

Our subgroup findings support personalized therapeutic paradigms. The lack of CSS benefit in N0-stage patients suggests biology-driven mechanisms treatment resistance, possibly linked to tumor immune microenvironment changes in elderly patients [37]. This observation aligns with translational findings from the LARCH study, which reported an inverse correlation between PD-L1 expression and CCRT efficacy in N0 NSCLC [38].

The SEER database provides valuable publicly accessible data, facilitating exploration of clinical issues. Nevertheless, our study had several limitations. Primarily, the database lacks detailed treatment information, including RT dosing, CT regimens, and immunotherapy specifics, limiting our ability to investigate the impact of these therapeutic factors. Additionally, the absence of data on treatment-related adverse effects restricted evaluation of therapy-induced complications. Finally, the lack of information on locoregional relapse and distant metastasis prevented assessment of locoregional relapse-free survival (LRRFS) and distant metastasis-free survival (DMFS).

Despite these constraints, our analysis provides population-level validation of NCCN guideline recommendations for fit elderly patients [39]. The SEER-derived evidence hierarchy (RT + CT superior to RT alone) remains valid even after adjusting for competing risks, supporting therapeutic intensification in carefully selected elderly patients. To address the identified critical gap in comorbidity assessment, a four-pillar research framework is proposed for future studies: (1) systematic geriatric evaluation using CCI and GA to stratify patient vulnerability; (2) prospective profiling of treatments, including radiation techniques (IMRT or proton therapy), chemotherapy tolerability, and CTCAE-graded toxicities; (3) multidimensional survival analysis integrating PROs, QoL metrics, and competing mortality risks; and (4) machine learning-based predictive modeling to develop comorbidity-adjusted therapeutic algorithms for elderly NSCLC patients.

## Conclusion

This study indicates that combining RT with CT, compared to RT alone, significantly improves OS and CSS in patients aged ≥65 years with newly diagnosed stage III inoperable NSCLC, based on data from the SEER-17 databases. Furthermore, patients at all stages except N0 may benefit from combined RT and CT. To confirm these findings, additional multicenter, prospective clinical trials are warranted.

## Supporting information

S1 Table (XLSX)

S2 Table (XLSX)
